# A Complete Audit Cycle of the Recording of the Baby's and Their Siblings' Age, Date of Births and Due Dates of Pregnant Mothers During the Initial Assessment Process for Patients Presenting to a Community Perinatal Mental Health Services

**DOI:** 10.1192/bjo.2024.564

**Published:** 2024-08-01

**Authors:** Chinenyenwa Eze, Emma Brotchie

**Affiliations:** Community Perinatal Mental Health Services, Heredfordshire and Worcestershire Health and Care NHS Trust, Worcester, United Kingdom

## Abstract

**Aims:**

- To find out the proportion of patients for whom the dates of births of their children, age and their due date were recorded during their initial assessment as a means of reducing risks through safeguarding.
- According to the Royal College of Psychiatrists: Standards for Community Perinatal Mental Health Services 5th Edition (2020), Under Section 5 – Rights, Infant Welfare and Safeguarding: during the initial assessment, the baby's age and date of birth and mother's due date should be recorded as part of the infants' physical and emotional care needs assessment.

**Methods:**

- All new patients discussed during multidisciplinary team meetings within a 2 month period from 01/08/2023 to 30/09/2023 were identified
- Their clinical records were audited.
- This information was cross-checked with the information provided on their referral letters.
- Patients attending preconception counselling were excluded.
- The initial results were presented in one of the multidisciplinary team meetings.
- The recording of the children's ages, date of birth or due dates of their mothers was re-audited two months later.

**Results:**

Audit

- A total of 70 new patients were discussed within the initial two months period.
- 25 out of the 70 (36%) did not attend their appointments and two patients (3%) cancelled their appointment.
- 1 patient who attended for preconception counselling was excluded.
- Of the remaining 42 patients that were assessed, 6 (14%) were primigravida while 36 (86%) patients were multiparous patients.
- 15 out of the 42 (36%) had their children's age, dates of birth and due date recorded while 27 out of the 42 (64%) lacked this record.

Re-audit

- A total of 65 patients were identified during the re-audit period
- 18 out of the 65 patients (28%) did not attend their appointment and one patient cancelled her appointment.
- One patient that attended for preconception counselling was excluded from the re-audit process.
- Of the remaining 45 patients that were assessed, 2 (4%) were primigravida and the remaining 43 (96%) were multiparous women.
- The age, dates of birth and the due date were recorded for 26 (58%) out of 45 patients while 19 out of the 45 patients (42%) did not have this record.

**Conclusion:**

- The children's ages were commonly recorded compared with their date of birth.
- Gestational ages of the pregnant mothers were commonly recorded compared with their due dates.
- Date of birth is needed for a quick check on a child for safeguarding reasons and this is useful during the admission of mothers onto a mother and baby unit.
- The re-audit showed a significant improvement in the documentation of this information in the patients' records.

